# Supplementary material for: Prevalence of COVID-19 and associated factors among healthcare workers in the war-torn Tigray, Ethiopia
Source: PLoS One. 2024 Nov 21;19(11):e0310128. doi: 10.1371/journal.pone.0310128 (PMC11581278; doi:10.1371/journal.pone.0310128)
Supplement: S2 Checklist — (DOCX) [file pone.0310128.s002.docx]

STROBE Statement—checklist of items that should be included in reports of observational studies

|  | Item No. | Recommendation | Page  No. | Relevant text from manuscript |
| --- | --- | --- | --- | --- |
| **Title and abstract** | 1 | Impact of war on COVID-19 infection among healthcare workers in Tigray, Northern Ethiopia | 1-2 |  |
|  |  | A facility based cross-sectional study was conducted to assess the prevalence of Coronavirus disease 2019 (COVID-19), using rapid diagnostic tests (RDTs), and its associated factors. The seroprevalence of COVID-19 among healthcare workers (HCWs) was 52.4% and 56.5% using Cellex and INNOVA antibody RDTs, respectively. The point prevalence using Abbot Antigen test was 14.2%. In the multivariate analysis, not having isolation area (AOR=19.6, 95% CI: 7.57, 50.78), re-using of personal protective equipment (PPE) (AOR=3.23, 95% CI: 1.54, 6.77), being symptomatic (AOE=2.4, 95% CI: 1.02, 5.67), and being a medical doctor, doctor of dental surgery and anesthetist (AOR=3.64, 95% CI: 1.05, 12.66) were significantly associated with having at least one positive result. | 25-44 |  |
| Introduction | | | |  |
| Background/rationale | 2 | Since the outbreak, Covid-19 has put an enormous encumbrance on healthcare system and healthcare workers (HCWs) worldwide [[6](#_ENREF_6)]. Their work characteristics exceptionally put them at a greater risk of acquiring the virus as they are at the frontline in fighting the outbreak. In a study done in United Kingdom and United States of America, there were 2,747 COVID-19 infected HCWs per 100,000, compared with 242/100,000 cases in the general public [[7](#_ENREF_7)]. The number of health professionals who succumb to the disease so far is not to be underestimated either. According to the world health organization, about 80-180 thousand HCWs have perished due to the virus from January 2020 to May 2021 [[8](#_ENREF_8)]. The brunt of the pandemic particularly falls on HCWs who live in war torn and besieged areas. COVID-19 mitigating programs and efforts started in Tigray, Ethiopia since the disease outbreak have been put in shambles as a result of a 2 year long war and siege [[10](#_ENREF_10)]. Attacks on health facilities and health workforce have resulted in disintegration of the health system in Tigray putting an enormous obstacle in implementing COVID-19 preventive measures. |  | 65-95 |
| Objectives | 3 | Although there have been concerns of increased transmission of the virus in Tigray since the eruption of the war, expressed by different bodies including UN agencies, the prevalence of the virus and its contributing factors among HCWs has not been studied during this cataclysmic period. Myriad of factors including breakdown in COVID-19 preventive measures, critically low vaccine coverage and dearth of supplies can put HCWs in the region at a considerable risk of acquiring the virus. Therefore, facility based cross-sectional study was conducted to assess the prevalence of COVID-19, using both antigen (Ag) and antibody (Ab) rapid diagnostic tests (RDTs), and its associated factors. This will help to see the burden of the problem for future COVID-19 prevention programs including vaccination. |  | 96-111 |
| Methods | | | |  |
| Study design | 4 | Cross-sectional study was done using Abbott antigen rapid diagnostics, **INNOVA and Cellex** lateral flow immune assay tests. | 150-161 |  |
| Setting | 5 | The study was conducted in two hospitals (Ayder comprehensive specialized hospital (ACSH) and Mekelle general hospital) in Mekelle town, Tigray, Ethiopia. Mekelle town is the capital city of Tigray. ACSH and Mekelle hospital give service to more than 9 million people with a catchment area involving Tigrai, Eritrea, Afar, and north western parts of Amhara region. The hospitals have more than 700 beds inpatient beds in four major departments and other specialty units. The two hospitals have the highest number of HCWs with 3000 and 500 in ACSH and Mekelle hospital, respectively. Data were collected from June 26 to December 30, 2021. | 114-126 |  |
| Participants | 6 | Information was obtained from registries of each hospital to identify whether the HCWs were vaccinated against COVID-19 or not. There was a total of 1,595 and 305 non-vaccinated HCWs in ACSH and Mekelle hospital, respectively. List of all non-vaccinated HCWs were identified to be included in the study and the vaccinated HCWs were excluded from the study. Samples were proportionally allocated among the two hospitals based on the number of unvaccinated HCWs (ACSH=(1,595/1,900)*317=274). Furthermore, the number of HCWs working at different units was considered for proper allocation of samples at each unit in each facility. HCWs working at COVID-19 isolation center, emergency outpatient department (OPD), intensive care unit (ICU), regular OPD, operation room (OR) and inpatient wards were selected from each hospital. After determining the allocated number of HCWs from each unit in each hospital, HCWs were randomly selected to participate in the current study. | 127-137 |  |
|  |  |  |  |  |
| Variables | 7 | The outcome variable of this study was COVID-19 infection using the different RDTs. Socio-demographic characteristics of HCWs, IPC practice, facility preparedness, and co-morbidities were used as explanatory variables.  **Acute/recent covid-19 infection in serologic tests:** When result was IgM positive or reactive; that is when only the M line developed in addition to the presence of C line.  **Previous/recent covid-19 infection in serologic tests:** When result was IgG positive or reactive; that is when only the M line developed in addition to the presence of C line.  **At least one positive test result:** having positive result for Cellex (Cel) IgM and/or Cel IgG and/or INNOVA IgM and/or IgG and/or Ag.  **Healthcare workers**: all paid and unpaid individuals who work in the healthcare setting who are at risk of both direct and indirect exposure to COVID-19 infected patients or their infectious secretions and materials (e.g., doctors, nurses, laboratory workers, facility or maintenance workers, cleaners, security guards, clinical trainees, volunteers etc).  **Aerosol generating procedures (AGPs)**: are procedures done in a hospital setting which generate aerosols when performed in a patient with respiratory illness such as COVID-19. The following procedures are usually considered AGPs (Intubation and extubation; Manual ventilation; Open suctioning; Cardiopulmonary resuscitation; Bronchoscopy and ENT fibreoptic endoscopy (unless carried out through a closed-circuit ventilation system); Dental procedures; Non-invasive ventilation; Continuous positive airway pressure ventilation; High frequency oscillatory ventilation; Induction of sputum). | 163-182 |  |
| Data sources/ measurement | 8* | Data were collected using interviewer guided questionnaire; and taking blood and nasopharyngeal samples from each of the selected HCWs. A structured questionnaire was used to get data on socio-demographic characteristics, IPC practice, facility preparedness, and co-morbidities from the participants while checklist was used to document the test results of three RDTs from the blood and nasopharyngeal samples. Two trained nurses and two laboratory technicians collected the data. All the required instructions and techniques of sampling and testing were followed by trained laboratory technicians. Pretest was done outside the selected hospitals prior to the data collection. | *141-161* |  |
| Bias | 9 | Multivariate analysis and Hosmer-Lemeshow goodness of fit test were used to address potential sources of bias | 185-192 |  |
| Study size | 10 | The estimated sample size was 326. It was determined using 26.1% as the prevalence of COVID-19 among HCWs, taken from a Zimbabwean study[[16](#_ENREF_16)], 95% confidence interval, 5% margin of error, and 10% non-response rate. | 123-126 |  |

Continued on next page

| Quantitative variables | 11 | Explain how quantitative variables were handled in the analyses. If applicable, describe which groupings were chosen and why |  |  |
| --- | --- | --- | --- | --- |
| Statistical methods | 12 | Descriptive statistics was used to analyze the characteristics of study participants and the magnitude of COVID-19 while bivariate and multivariate logistic regression was applied to assess the association between dependent and independent variables. Pearson Chi-square test was also used to see relationship between variables. Variables with p-value < 0.2 in the bivariate analysis were chosen to be analyzed in multivariate analysis to minimize the effect of confounding factors between variables. The presence and strength of association was determined using odds ratio with 95% confidence interval. Variables with P value < 0.05 were assumed as statistically significant. | 184-191 |  |
| Results | | | | |
| Participants | 13* | A total of 326 HCWs were eligible to be included in the study but 9 HCWs refused to give nasopharyngeal and blood samples; hence they were excluded in the analysis. | 204 |  |
| Descriptive data | 14* | The rate of COVID-19 infection was 70%, 67% and 63% among HCWs with diabetes, Asthma and cardiovascular disorder, respectively. The seroprevalence of COVID-19 among HCWs was 52.4% (166) and 56.5% (179) using Cellex and INNOVA Ab RDTs, respectively. The rate of acute/recent infection using INNOVA and Cellex Ab RDTs was 22.7% (72) and 6% (19), respectively. A total of 150 (47.3%) and 159 (50.2%) HCWs were positive for INNOVA IgG and Cel IgG, respectively. The point prevalence of COVID-19 using Abbott Ag test was 14.2% (45). Totally, 207 (65.3%) of the HCWs had at least one positive result indicating very high prevalence of COVID-19 infection among HCWs in the health facilities included in the study. Rate of infection was higher among males than females in Ab RDTs, but it was not statistically significant in any of the tests (P-value>0.05). Less than half (131 (41.3%)) of study participants received IPC training. Most (191 (60.3%)) of the HCWs re-used PPE in their daily activities. Study participants were asked if their hospital had isolation centre for contact of, suspected and confirmed COVID-19 cases during the time of testing and 180 (56.8%) of them responded positively. Only 131 (41.3%) reported their facility had triaging system for COVID-19 cases during the time of testing. All HCWs in this study perceived that their health facilities did not have separate laundry area and waste disposal system for COVID-19 cases; and no separate residence for HCWs who worked in areas isolated for COVID-19 quarantined, suspected and confirmed cases. | 204-321 |  |
| Outcome data | 15* | COVID-19 infection | *331* |  |
| Main results | 16 | In the multivariate analysis, not having isolation area for contacts of, suspected and confirmed COVID-19 cases in the health facility (AOR=**19.6, 95% CI: 7.57, 50.78)**, reusing of PPE (AOR=**3.23, 95% CI: 1.54, 6.77)**, having symptoms of COVID-19 within 14 days of testing (AOE=**2.4, 95% CI: 1.02, 5.67),** and being MD, doctor of dental surgery (DDS) and anaesthetist (AOR=**3.64, 95% CI: 1.05, 12.66)** were significantly associated with having at least one positive result. Moreover, having history of contact with suspected or confirmed COVID-19 individuals within 14 days of testing (AOR=**2.96, 95% CI: 1.09, 8.0)** was significantly associated with being positive for Abbott Ag test while receiving IPC training (AOR=**0.24, 95% CI: 0.07, 0.85)** conferred protection. Wearing mask when out in the community was protective against having Cel IgM and/or INNOVA IgM positive results (AOR=**0.32, 95% CI: 0.15, 0.68).** In addition, age 35-44 (AOR=**2.69, 95% CI: 1.46, 4.93)** and age>45 (AOR= **3.31, 95% 1.04, 10.54)** were significantly associated with having INNOVA IgG and/or Cel IgG positive results while working in units that offer masks to quarantined, suspected or confirmed COVID-19 cases (AOR=**0.31, 95% CI: 0.15, 0.67)** was protective. | 332-346 |  |

Continued on next page

| Discussion | | | | |
| --- | --- | --- | --- | --- |
| Key results | 18 | We found that the rate of COVID-19 infection among HCWs in the war wracked region of Tigray was high across all tests; and there was variation in the prevalence of the infections using the different tests. The concordance rate of symptoms and positivity in the tests that detect active infection also showed disparity; with INNOVA IgM having the lowest concordance rate. Four sets of factors were associated with having COVID-19 infection. These findings bring to light how HCWs practicing in fragile regions, where disruption of health sector is ubiquitous, are affected with the COVID-19 pandemic. Factors related to sociodemographic characteristics, IPC practice and facility preparedness, exposure risk, and clinical characteristics contributed to the increased prevalence of infection with SARS-COV-2. | 362-371 |  |
| Limitations | 19 | Since nucleic acid amplification tests (NATs) were not available in the region during the study period, we were not be able to assess the specificity and sensitivity of the RDTs. | 471-473 |  |
| Interpretation | 20 | In this study, the prevalence of COVID-19 among HCWs was among the highest in the world. Inability to provide training to HCWs on the preventive measures of COVID-19, shortage of PPE supply, difficulty in sustaining facility preparedness at the level required, and low vaccination coverage in the region have contributed to the high prevalence of the infection observed during this cataclysmic period. Therefore, we recommend for the responsible bodies including international organizations to increase supply of PPE, escalate trainings on COVID-19 preventive measures, provide vaccines, and help re-establish triaging systems and isolation centres in the studied facilities in particular and in the region in general. | 477-484 |  |
| Generalisability | 21 | The results discern contributing elements that need intervention to halt COVID-19 transmission in the health facilities in particular and in the community at large in war torn regions as high prevalence in health facilities can be a snapshot of what is happening in the community; and as the virus can gain a foothold in these regions paving the way for future waves of the pandemic. | 371-375 |  |
| Other information | |  | | |
| Funding | 22 | No funding was acquired for this study. |  |  |

*Give information separately for cases and controls in case-control studies and, if applicable, for exposed and unexposed groups in cohort and cross-sectional studies.

**Note:** An Explanation and Elaboration article discusses each checklist item and gives methodological background and published examples of transparent reporting. The STROBE checklist is best used in conjunction with this article (freely available on the Web sites of PLoS Medicine at http://www.plosmedicine.org/, Annals of Internal Medicine at http://www.annals.org/, and Epidemiology at http://www.epidem.com/). Information on the STROBE Initiative is available at www.strobe-statement.org.
