# Supplementary material for: Prevalence of COVID-19 and associated factors among healthcare workers in the war-torn Tigray, Ethiopia
Source: PLoS One. 2024 Nov 21;19(11):e0310128. doi: 10.1371/journal.pone.0310128 (PMC11581278; doi:10.1371/journal.pone.0310128)
Supplement: S1 File — (DOCX) [file pone.0310128.s003.docx]

# Supplementary

**S1 table.** **Socio-demographic characteristics of study participants, Tigray, Ethiopia, 2021 (n=317).**

| **Socio-demographic variables** | **Frequency** | **Percentage** |
| --- | --- | --- |
| **Median age** | 30 years (IQR:27-35), Min=19 & Max=77) | |
| **Age in years** |  |  |
| <=24 | 40 | 12.6 |
| 25-34 | 184 | 58.0 |
| 35-44 | 66 | 20.8 |
| >=45 | 27 | 8.5 |
| **Sex** |  |  |
| Male | 131 | 41.3 |
| Female | 186 | 58.7 |
| **Religion** |  |  |
| Orthodox Christian | 309 | 97.5 |
| Protestant | 4 | 1.3 |
| Catholic | 2 | 0.6 |
| Muslim | 2 | 0.6 |
| **Marital status** |  |  |
| Single | 127 | 40.1 |
| Married | 167 | 52.7 |
| Divorced | 23 | 7.3 |
| **Educational status** |  |  |
| Primary school and below | 16 | 5.0 |
| Secondary school | 23 | 7.3 |
| Diploma | 59 | 18.6 |
| Degree | 161 | 50.6 |
| Master and MD | 58 | 18.3 |
| **Profession** |  |  |
| Nurse and midwife | 124 | 39.1 |
| MD, DDS, ANT^a^ | 55 | 17.4 |
| Other health professionals ^b^ | 40 | 12.6 |
| Supportive (Janitors) | 22 | 6.9 |
| Other supportive staffs ^c^ | 76 | 24.0 |

a=Medical doctor, Doctor of Dental surgery, Anesthetist, b= Laboratory technician/technologist, pharmacist, radiology technician/technologist; c= Casher, security, morgue attendant, porter, oxygen attendant, social worker, maintenance worker, hospital data encoder.

**S2 table.** **Healthcare workers’ infection prevention and control practice to SARS-CoV-2 infection, Tigray, Ethiopia, 2021 (n=317).**

| **IPC practice** | **Frequency** | **Percentage** |
| --- | --- | --- |
| **IPC training** |  |  |
| Yes | 131 | 41.3 |
| No | 186 | 58.7 |
| **Types of standard precautions usually practiced at work** |  |  |
| Hand hygiene | 277 | 87.4 |
| Respiratory hygiene | 50 | 15.3 |
| Gloves/utility gloves | 131 | 41.3 |
| Medical gown | 125 | 39.4 |
| Eye wear | 19 | 6.0 |
| Surgical mask | 283 | 89.3 |
| **Types of PPE used for transmission-based precautions** |  |  |
| Surgical mask | 307 | 96.8 |
| Eye goggle/face shield | 22 | 6.9 |
| Gloves/utility gloves | 113 | 35.6 |
| Long sleeved gown/Coverall | 21 | 6.6 |
| Respiratory (N95/FFP3) | 44 | 13.9 |
| Shoe cover/Boots | 28 | 8.8 |
| Hair cover | 24 | 7.6 |
| **AGP within 14 days of testing** |  |  |
| Yes | 29 | 9.1 |
| No | 288 | 90.9 |
| **Types of AGP performed** |  |  |
| Aspiration/open suctioning of respiratory secretions | 15 | 51.7 |
| Intubation | 5 | 17.2 |
| Cardio-pulmonary resuscitation (CPR) | 13 | 44.8 |
| Bronchoscopy | 1 | 3.4 |
| Aerosolized nebulizer | 0 | 0 |
| Non-invasive ventilation | 0 | 0 |
| High-flow oxygen | 2 | 6.9 |
| **Used N95/FFP3 while performing AGP** |  |  |
| Yes | 0 | 0 |
| No | 29 | 100 |
| **Re-used PPE** |  |  |
| Yes | 191 | 60.3 |
| No | 126 | 39.7 |
| **Types of re-used PPE** |  |  |
| Medical mask/surgical mask | 184 | 96.3 |
| Respiratory (N95/FFP3) | 22 | 11.5 |
| Eye goggle/face shield | 3 | 1.6 |
| Medical gown | 51 | 26.7 |
| Surgical gown | 7 | 3.7 |
| Locally made gown | 3 | 1.6 |
| Gloves (utility) | 10 | 5.2 |
| Shoe cover/boots | 9 | 4.7 |
| Hair cover | 3 | 1.6 |
| **Practicing social distancing** |  |  |
| Yes | 37 | 11.7 |
| No | 280 | 88.3 |
| **Wearing mask when out in the community** |  |  |
| Yes | 119 | 37.5 |
| No | 198 | 62.5 |
| **Types of mask wore in the community** |  |  |
| Medical mask | 118 | 99.2 |
| N95 | 5 | 4.2 |
| Cloth mask | 16 | 13.4 |
| **Hand hygiene out of health facility** |  |  |
| Yes | 148 | 46.8 |
| No | 169 | 53.3 |
| **Respiratory hygiene out of health facility** |  |  |
| Yes | 47 | 14.8 |
| No | 270 | 85.2 |

**S3**: Factors associated with positive Abbot antigen test result among healthcare workers in Tigray, Ethiopia (n=317).

| **Variables** | **COVID-19** | | **COR (95%, CI)** | **AOR (95%, CI)** |
| --- | --- | --- | --- | --- |
|  | **Yes (%)** | **No (%)** |  |  |
| **Occupation** |  |  |  |  |
| Nurse or Midwife | 18 (14.5) | 106 (85.5) | 1 | 1 |
| MD, ANT, DDS | 19 (34.5) | 36 (65.5) | **3.11 (1.47, 6.56)**** | **4.51 (1.58, 17.23)*** |
| Other Health professionals | 1 (2.5) | 39 (97.5) | 0.15 (0.02, 1.17) | 0.27 (0.03, 2.86) |
| Supportive (Janitors) | 4 (18.2) | 18 (81.8) | 1.31 (0.4, 4.31) | 1.27 (0.25, 6.56) |
| Other Supportive staffs | 3 (3.9) | 73 (96.1) | **0.24 (0.07, 0.85)*** | 0.29 (0.06, 1.34) |
| **IPC training** |  |  |  |  |
| Yes | 10 (8.5) | 107 (91.5) | **0.44 (0.21, 0.93)*** | **0.24 (0.07, 0.85)*** |
| No | 35 (17.5) | 165 (82.5) | 1 | 1 |
| **Isolation area** |  |  |  |  |
| Yes | 8 (5.1) | 149 (94.9) | **0.18 (0.08,0.4)***** | 0.9 (0.27, 3.04) |
| No | 37 (23.1) | 123 (76.9) | 1 | 1 |
| **AGP procedures within 14 days of testing** |  |  |  |  |
| Yes | 15 (51.7) | 14 (48.3) | **9.21 (4.07, 20.93)***** | 3.45 (0.83, 14.42) |
| No | 30 (10.4) | 258 (89.6) | 1 | 1 |
| **Re-used PPE** |  |  |  |  |
| Yes | 39 (19.3) | 163 (80.7) | **4.35 (1.78, 10.62)***** | 2.41 (0.66, 8.81) |
| No | 06 (5.2) | 109 (94.8) | **1** | 1 |
| **Contact with suspected and/or confirmed COVID-19 cases** |  |  |  |  |
| Yes | 34 (30.9) | 76 (69.1) | **7.97 (3.84, 16.53)***** | **2.96 (1.09, 8.0)*** |
| No | 11 (5.3) | 196 (94.7) | **1** | 1 |
| **Symptoms of COVID-19 within 14 days of testing** |  |  |  |  |
| Yes | 41 (45.5) | 49 (54.5) | **46. 7 (15.97, 136.3)***** | **67.1 (16.41, 274.3)***** |
| No | 4 (1.8) | 223 (98.2) | **1** | **1** |

*p-value <0.05; **p-value 0.01-0.002; ***p-value <=0.001

**S4**: Factors associated with INNOVA and/or Cellex IgM positive result among healthcare workers in Tigray, Ethiopia, 2021 (n=317).

| **Variables** | **COVID-19** | | **COR (95%, CI)** | **AOR (95%, CI)** |
| --- | --- | --- | --- | --- |
|  | **Yes (%)** | **No (%)** |  |  |
| **IPC training** |  |  |  |  |
| Yes | 15 (12.8) | 102 (87.2) | **0.37 (0.20, 0.69)**** | 0.49 (0.24, 1.02) |
| No | 57 (28.5) | 143 (71.5) | 1 | 1 |
| **Unit offers masks to quarantined, suspected or confirmed COVID-19 patients** |  |  |  |  |
| Yes | 8 (12.3) | 56 (87.7) | **0.42 (0.19, 0.93)*** | 0.99 (0.39, 2.51) |
| No | 64 (25.3) | 189 (74.7) | 1 | 1 |
| **Re-used PPE** |  |  |  |  |
| Yes | 57 (28.2) | 145 (71.8) | **2.62 (1.41, 4.89)**** | 1.17 (0.55, 2.49) |
| No | 15 (13.0) | 100 (87.0) | 1 | 1 |
| **Contact with suspected and/or confirmed COVID-19 cases** |  |  |  |  |
| Yes | 36 (30.9) | 74 (69.1) | **2.31 (1.35, 3.95)**** | 1.54 (0.83, 2.85) |
| No | 36 (17.4) | 171 (82.6) | **1** | 1 |
| **Wearing maskin the community** |  |  |  |  |
| Yes | 11 (9.2) | 108 (90.8) | 0.23 (0.11,0.46)*** | **0.32 (0.15, 0.68)**** |
| No | 61 (30.8) | 137 (69.2) |  | 1 |
| **Symptoms of COVID-19 with in the past 14 days of data collection period** |  |  |  |  |
| Yes | 35 (38.8) | 55 (61.2) | **3.27 (1.88, 5.67)***** | **2.69 (1.46, 4.93)***** |
| No | 37 (16.3) | 190 (83.7) | **1** | **1** |
| **Working hours per day** |  |  |  |  |
| <=8hrs | 62 (21.1) | 232 (78.9) | **0.35 (0.15, 0.83)*** | 0.60 (0.22, 1.63) |
| >8hrs | 10 (43.5) | 13 (56.5) | 1 | 1 |

*p-value <0.05; **p-value 0.01-0.002; ***p-value <=0.001

**S5**: Factors associated with INNOVAand/or Cellex IgGpositive result among healthcare workers in Tigray, Ethiopia (n=317).

| **Variables** | **COVID-19** | | **COR (95%, CI)** | **AOR (95%, CI)** |
| --- | --- | --- | --- | --- |
|  | **Yes (%)** | **No (%)** |  |  |
| **Age in year** |  |  |  |  |
| <=24 | 17 (42.5) | 23 (57.5) | 1 | 1 |
| 25-34 | 91 (49.5) | 93 (50.5) | 1.32 (0.66, 2.64) | 1.80 (0.82, 3.93) |
| 35-44 | 41 (62.1) | 25 (37.9) | 2.22 (1.00, 4.49) | **2.88 (1.16, 7.13)*** |
| >=45 | 19 (73.1) | 08 (26.9) | **3.21 (1.14, 9.06)*** | **3.31 (1.04, 10.54)*** |
| **IPC training** |  |  |  |  |
| Yes | 36 (27.7) | 81 (72.3) | **0.23 (0.14, 0.37)***** | **0.40 (0.22, 0.72)**** |
| No | 132 (66.0) | 68 (34.0) | 1 | **1** |
| **Unit offers masks to quarantined, suspected or confirmed COVID-19 patients** |  |  |  |  |
| Yes | 13 (20.3) | 51 (79.7) | **0.16 (0.08, 0.31)***** | **0.31 (0.15, 0.67)**** |
| No | 155 (61.3) | 98 (38.7) | 1 | 1 |
| **Re-used PPE** |  |  |  |  |
| Yes | 135 (66.8) | 67 (33.2) | **5.0 (3.04, 8.25)***** | **2.72 (1.51, 4.91)***** |
| No | 33 (28.7) | 82 (71.3) | **1** | **1** |
| **Wearing mask when out in the community** |  |  |  |  |
| Yes | 50 (42.0) | 69 (58.0) | **0.49 (0.31, 0.78)***** | 0.94 (0.53, 1.69) |
| No | 118 (59.6) | 80 (40.4) | **1** | 1 |
| **Work unit** |  |  |  |  |
| COVID-19 isolation center | 10 (41.7) | 14 (58.3) | 0.71 (0.29, 1.77) | 1.12 (0.38, 3.30) |
| EM and OPD | 99 (60.7) | 64 (39.3) | 1.55 (0.93, 2.57) | 1.08 (0.59, 1.96) |
| ICU and OR | 11 (32.3) | 23 (67.7) | 0.48 (0.21, 1.09) | 0.44 (0.17, 1.11) |
| Wards | 48 (50.0) | 48 (50.0) | 1 | 1 |

*p-value <0.05; **p-value 0.01-0.002; ***p-value <=0.001

**S6 table. Relationship between occupation and history of contact among healthcare workers in Tigray, Ethiopia, 2021 (n=317).**

|  | | Contact with COVID-19 confirmed or suspected cases within 14 days of testing | | Total | p-value |
| --- | --- | --- | --- | --- | --- |
|  |  | Yes | No |  |  |
| Occupations | Nurse or Midwife | 41 (33.1%) | 83 (66.9%) | 124 (100%) | 0.001 |
|  | MD, ANT, DDS | **32 (58.2%)** | 23 (41.8%) | 55 (100%) |  |
|  | Other health professionals | 10 (25.0%) | 30 (75.0%) | 40 (100%) |  |
|  | Supportive staff (Janitors) | 7 (31.8%) | 15 (68.2%) | 22 (100%) |  |
|  | Other Supportive staffs | 20 (26.3%) | 56 (73.7%) | 76 (100%) |  |
| Total | | 110 (34.7%) | 207 (65.3%) | 317 |  |

*p-value using Pearson Chi-square test*
