# Supplementary material for: Prevalence of COVID-19 and associated factors among healthcare workers in the war-torn Tigray, Ethiopia
Source: PLoS One. 2024 Nov 21;19(11):e0310128. doi: 10.1371/journal.pone.0310128 (PMC11581278; doi:10.1371/journal.pone.0310128)
Supplement: Attachment — Submitted filename: PONE34484.docx [file pone.0310128.s004.docx]

Editor comments

1. You submitted research entitled as “Impact of war on COVID-19 infection among healthcare workers in Tigray, Northern Ethiopia”. The study was conducted using a cross-sectional study design which lasted about five months. The assessment of impact requires data for a long period of years. However, in your study, you haven’t mentioned anything about the effects of COVID-19 on HCWs. It begins and ends with prevalence and its associated factors. Besides, there is no single question that is targeted to assess the effect/impact. Thus, you are recommended to modify your title “Prevalence of COVID-19 and associated factors among HCWs in Tigray, Ethiopia” as it could fit to your findings.
2. It is essential to differentiate whether it was war or conflict in Tigray region during the study period. Is there formally declared for war at the time of your data collection? Sensitive issues seek evidence. Thus, you are requested to modify with the available evidence.
3. COVID-19 is an infectious respiratory disease. In the acronym, COVID-19, “I” stands for Infectious. Thus, no need to use it repeatedly.
4. When you mention authors affiliation it is suggested to state from the specific unit to the general unit (For instance: department of…, school/college, institute/…, university, region, country). Do not intermix.
5. The corresponding author is the main author who communicate with the journal regarding your work. You mentioned all of your members as the corresponding author as seen in the first page of your manuscript. Please try to adhere to the journal guideline.
6. English language usage still need great effort.
7. The scientific reasonings in the discussion section and the methods needs revision.
