# Supplementary material for: Prevalence of COVID-19 and associated factors among healthcare workers in the war-torn Tigray, Ethiopia
Source: PLoS One. 2024 Nov 21;19(11):e0310128. doi: 10.1371/journal.pone.0310128 (PMC11581278; doi:10.1371/journal.pone.0310128)
Supplement: Attachment — Submitted filename: POINTB~1.DOC [file pone.0310128.s005.DOC]

Dear editors and reviewers

First of all, we would like to thank you very much for the chance you gave us to revise the manuscript. We are very pleased to know that our manuscript was rated as potentially acceptable for publication in PLOS, subject to a radical revision and response to the comments raised by the editor and the reviewer.

Based on the instructions provided in the journal's website and your email on May 15, 2023, we attached the file of our revised manuscript word (doc.). We have revised the manuscript along the line of all comments made by the editor and the referee.

Appended to this letter is our point-by-point response to the comments raised by the referee and the editor.

Sincerely,

Bisrat, MD, Internist.

**Response to editor and reviewer**

A. **Comments from the editor**
1. You submitted research entitled as “Impact of war on COVID-19 infection among healthcare workers in Tigray, Northern Ethiopia”. The study was conducted using a cross-sectional study design which lasted about five months. The assessment of impact requires data for a long period of years. However, in your study, you haven’t mentioned anything about the effects of COVID-19 on HCWs. It begins and ends with prevalence and its associated factors. Besides, there is no single question that is targeted to assess the effect/impact. Thus, you are recommended to modify your title “Prevalence of COVID-19 and associated factors among HCWs in Tigray, Ethiopia” as it could fit to your findings.

**Response:**

Thank you very much for your invaluable comments. We accept your suggestion and we have changed the title to “Prevalence of COVID-19 and associated factors among HCWs in the war-torn Tigray, Ethiopia.” Please see the title in the revised manuscript with track changes. We are open for further suggestions.

2. It is essential to differentiate whether it was war or conflict in Tigray region during the study period. Is there formally declared for war at the time of your data collection? Sensitive issues seek evidence. Thus, you are requested to modify with the available evidence.

**Response:**

Thank you for raising this comment. The data was collected during the second phase of the fighting where the war had already spilled over to other regions of Ethiopia. According to a book by Dan smith, war is defined as “an armed conflict involving at least two or more centrally organized parties, in disputes about power over government and territory, engaging in armed conflict in formal military formation that have caused at least the death of 1000 people.”(<https://berghof-foundation.org/library/trends-and-causes-of-armed-conflict>) The fight betweenTigray region on the one end and the Federal government of Ethiopia, regional special forces and Eritrea on the other end has resulted in the death of 100s of thousands of people.In addition, different institutional bodies, including the European parliament, have addressed the situation as war. (<https://news.un.org/en/story/2022/09/1127481>, <https://www.europarl.europa.eu/thinktank/en/document/EPRS_BRI(2022)739244>.

We are open for further comments and suggestions.

3. COVID-19 is an infectious respiratory disease. In the acronym, COVID-19, “I” stands for Infectious. Thus, no need to use it repeatedly.

**Response:**

Thank you again. We have removed it from statements where we put it next to “COVID-19”. Please see the amendment in the revised manuscript with track changes.

4. When you mention authors affiliation it is suggested to state from the specific unit to the general unit (For instance: department of…, school/college, institute/…, university, region, country). Do not intermix.

**Response**:

Thank you for this constructive comment. We have put the authors’ affiliations according to your suggestion. Please see the amendment in the revised manuscript with track changes.

5. The corresponding author is the main author who communicate with the journal regarding your work. You mentioned all of your members as the corresponding author as seen in the first page of your manuscript. Please try to adhere to the journal guideline.

**Response**:

Thank you for this constructive comment. The corresponding author of this manuscript is Dr. Bisrat Tesfay Abera. We have removed the other authors from the corresponding author list. Please see the amendment in the revised manuscript with track changes.

6. English language usage still need great effort.

**Response:**

Thank you again for your constructive comment.

To the best of our knowledge, we have tried to improve the language usage. Please see the amendment in the revised manuscript with track changes.

7. The scientific reasonings in the discussion section and the **methods** needs revision.

**Response:**

Thank you again for your constructive comment.

To the best of our knowledge, we have tried to revise the methods and discussion. Please see the amendment in the revised manuscript with track changes.

B. **Reviewer comments**
8. Abstract
   • Line 30—revise the statement “ ….. the association between dependent and independent variables” to “…. factors affecting COVID-19 infection among health workers”.

**Response:**

Thank you for the invaluable and constructive comments. We have changed the statement as per your suggestion. Please see the revision in the updated manuscript with track changes.
• Line 31-- Results: while the title mentions conflict, I don’t see any key word stating conflict. Any idea for the authors on this issue?

**Response**:

Thank you for raising this concern. As per the editor’s suggestion, we agreed to change the title and removed the “impact of the war” from the title. But, to not to completely ignore the contribution of the war, we have added the phrase “in the war-torn region of Tigray.” We are ready for further suggestions and comments. Please see the change we have made in the revised manuscript with track changes.

**Introduction**
• Lines 80-86: Any data that conflict causes extra covid-19 infection, mortality and other related complications?

**Response**:

Thank you for the suggestion again. We have incorporated studies from Yemen. Please see the revision made in the manuscript with track changes.

• Line 90: citations needed

**Response:**

Thank you for your input. We accept your suggestion and we have added a citation. Please see the revision in the manuscript with track changes.

• Line 95—remove the subtitle ‘local context’, and cut and paste the points about local vaccine coverage from lines 105-11 to after line 95.

**Response:**

Thank you for your suggestion. We have removed the subtitle and moved the content about the vaccine coverage in Tigray to the recommended part of the introduction. please look in the manuscript with the track changes.

• NB. Could the authors make ‘Tigrai/y’ consistent? Perhaps use Tigray throughout?

**Response:**

We acknowledge your comment and have tried to be consistent with the word Tigray.

• In rationale of the study, the authors need to put their hypothesis and contextualized to conflict. For example, they could describe how the war in Tigray caused increased incidence and prevalence of illnesses (e.g. HIV infection), mortality and loss-to follow up (e.g. hypertension), and others. And covid-19 infection could also be hypothesized that theincidence among HWs will be high. This way it can be contextualized to the conflict.

**Response:**

Thank you again for this important insight. We have added the effect of the war on HIV service delivery and care of patients with hypertension in Tigray, and to the best of our knowledge, we have tried to contextualize it to the conflict. Please see the revised manuscript with track changes.

• Line 123: Why are study participants unvaccinated? I think it’s important to consider the case in all health workers so that even vaccine effectiveness among health workers could be described. Could the authors put their rationale why they only included mention unvaccinated health works in the method section but also add a limitation at the end of discussion that they could have included vaccinated HWs and even saw how the effectiveness of the vaccine among those vaccinated/unvaccinated?

**Response:**

Thank you again. Since we used serology tests to assess the prevalence of COVID-19 among healthcare workers, we excluded HCWs who were vaccinated to avoid cross reactivity and minimize false positivity. We accept your suggestion and have included the reasons why we only considered the unvaccinated HCWs in the methods section. We have also considered it as a limitation for not including the vaccinated HCWs at the end of the discussion section. Please see the manuscript with track changes. ([Considerations for the use of antibody tests for SARS CoV-2 - first update (europa.eu)](https://www.ecdc.europa.eu/sites/default/files/documents/Considerations-for-the-use-of-antibody-tests-for-SARS-CoV2-first-update.pdf))
**Results**
• Line 332/3: I wonder why the CI is very high for the variable suspected and confirmed COVID-19 cases in the health facility (AOR=19.6, 95% CI: 7.57, 50.78).

**Response:**

Thank you for the comment. In table 5 of the result section, the variable “Contact with suspected and/or confirmed COVID-19 case” is not significant at multivariate analysis, only significant at bivariate analysis where there was higher risk of infection among HCWs who had contact with suspected/confirmed COVID-19 case (COR=1.92, 95% CI: 1.15, 3.2). The high CIs was for the variable “Isolation area” (AOR=19.6, 95% CI: 7.57, 50.78). This indicates those HCWs who work outside the COVID-19 isolation area were more likely to experience COVID-19 infection. HCWs who work at isolation center might have more awareness about the disease and might have received more training compared to those HCWs working in other units. Regarding the high CI; it could be due to the small sample size in the cells; of the four cells, one cell has 08 samples. This may lead to wide CI and higher odds ratio.

• While the result is presented very well, I still wonder how the title was contextualized to the conflict in Tigray --- the authors need to add some contextualized concept.

**Response:**

Thank you for this critical input. As mentioned above, we have revised the title as we did not have data to compare the difference in prevalence of COVID-19 before the start of the war and during the war. We are open for further suggestions and comments.

**Discussion**
• Line 377-379. I think the comparison should also be done with other conflict-affected areas

**Response**:

Thank you again. We accept the recommendation and we have added data from Yemen. Please see the revision in the revised manuscript with track changes.

• Could the authors compare if the incidence among the health workers was different when compared to the general population in the region? And how was the incidence when compared to the incidence before the start of the conflict in the region?

**Response:**

Thank you for this recommendation. We have managed to get data about the incidence of the infection in the general population before the war and we have included it in the discussion. Unfortunately, we were not able to find data about the incidence of COVID-19 in the general population during the war. Please see the revision in the revised manuscript with track changes.

• Add the above mentioned limitation

**Response:**

Thank you very much. We accept your suggestion and we have put it as one of the limitations of the study. Please see the revision in the revised manuscript with track changes.
NB. The paper needs heavy English proof.

**Response:**

Thank you very much. We accept the recommendation and have done the English proofing. Please see the manuscript with track changes.
