# Supplementary material for: Prevalence of COVID-19 and associated factors among healthcare workers in the war-torn Tigray, Ethiopia
Source: PLoS One. 2024 Nov 21;19(11):e0310128. doi: 10.1371/journal.pone.0310128 (PMC11581278; doi:10.1371/journal.pone.0310128)
Supplement: Attachment — Submitted filename: REVIEW OF ARTICLE covid19 and Tigray War.docx [file pone.0310128.s006.docx]

REVIEW OF ARTICLE

TITLE : Prevalence of COVID-19 and associated factors among healthcare workers in the war-torn Tigray, Ethiopia.

Abstract

Results

Line 38- 40 : The confidence Interval should be written in form of range. Example, 7.57- 50.78

Introduction

Page 4,line 91: … existing COVID-19 surveillance NOT existed COVID-19 surveillance

Methodology

Page 6, line 118: can be revised to “ The hospitals have more than 700 in-patient beds…” rather than “The hospitals have more than 700 beds inpatients beds …”

Data analysis

Page 9, line 192: Hosmer-Lemeshow goodness of fit test was run .. √

Hosmer-Lemeshow goodness of fittest was ran… X

Results

It is important that the author should start by briefly telling us how the 97.2% response rate was arrived at.

Page 10, line 206, should be written as “ … average age was 32.0 ± 8.4 years.”

Discussion

Page 20,line 385: Insert” likely” between **the** and **reasons =** the likely reasons …

Page 20, line 398: this finding is in line with what… √

This finding is in line to what … X

Page 22, line 445

This could be explained by the difference in degree … √

This could be explianed by the deference in degree … X

References

Line 513, reference 6: Lapolla PMA √

Lapolla P, M.A. X

Line 520, reference 8: There should be full-stop after the author’s name, that is, after WHO.

The days for which the literature was cited online should be written.

Line 569, reference 24: Insert fullstop after the name of author, ie CDC.

Line 588, reference 30: There should be up to 6 authors spelt out before stating et al.
