# Supplementary material for: Prevalence of COVID-19 and associated factors among healthcare workers in the war-torn Tigray, Ethiopia
Source: PLoS One. 2024 Nov 21;19(11):e0310128. doi: 10.1371/journal.pone.0310128 (PMC11581278; doi:10.1371/journal.pone.0310128)
Supplement: Attachment — Submitted filename: Response to Reviewers.doc [file pone.0310128.s007.doc]

**May 20, 2024**

**Dear editors**

First of all, we would like to thank you very much for the chance you have given us to revise the manuscript. We are very pleased to know that our manuscript was rated as potentially acceptable for publication in PLOS ONE, subject to revision and response to the comments raised by the editor and the reviewers.

Based on the instructions provided in the journal's website and your email on May 14, 2024, we have attached the file of our revised manuscript with track changes, the clean version and response to reviewers. We have revised the manuscript along the line of all comments made by the editor and reviewers.

Appended to this letter is our point-by-point response to the comments raised by reviewers.

Sincerely,

Bisrat, MD, Internist.

Mekelle University, College of Health Sciences, Tigray, Ethiopia.

**Response to editor**

Thank you for your invaluable comments!

We have corrected the grammatical error of many sentences according to the suggestions by the editor and reviewers. We have tried our best to proof-read the language. We have made revisions to the manuscript regarding choice of words, typos, grammar and consistency issues.

To the best of our knowledge, we have reviewed the reference list and have ensured that it is complete and correct. We have not cited papers that have been retracted.

**Response to reviewers**

A. **Comments from Reviewer 1**
1. **Abstract**

**Results: Line 38- 40: The confidence Interval should be written in form of range. Example, 7.57- 50.78**

**Response:**

Thank you very much for your invaluable comments! We accept your suggestion and we have modified it accordingly. All confidence interval throughout the mnusacripte written in form of range. Please see the title in the revised manuscript with track changes.

2. **Introduction**

**Page 4, line 91: … existing COVID-19 surveillance NOT existed COVID-19 surveillance**

**Response:**

Thank you! We have rearranged the sentence according to your suggestion. Please see the title in the revised manuscript with track changes.

3. **Methodology
   Page 6, line 118: can be revised to “The hospitals have more than 700 in-patient beds…” rather than “The hospitals have more than 700 beds inpatients beds …”**

**Response**:

Thank you for this constructive comment. We have amended the sentence according to your suggestion. Please see the amendment in the revised manuscript with track changes.

4. **Data analysis
   Page 9, line 192: Hosmer-Lemeshow goodness of fit test was run .. √
   Hosmer-Lemeshow goodness of fittest was ran… X**

**Response**:

Thank you again! We have revised it in line to your suggestion.Please see the amendment in the revised manuscript with track changes.

5. **Results
   It is important that the author should start by briefly telling us how the 97.2% response rate was arrived at.
   Page 10, line 206, should be written as “… average age was 32.0 ± 8.4 years.”**

**Response:**

Thank you again for your constructive comment! We have included a sentence about how the 97.2% was arrived. We have also corrected line 206 according to your suggestion. Please see the amendment in the revised manuscript with track changes.

6. **Discussion**

**Page 20, line 385: Insert” likely” between the and reasons = the likely reasons …
Page 20, line 398: this finding is in line with what… √
This finding is in line to what … X
Page 22, line 445
This could be explained by the difference in degree … √
This could be explianed by the deference in degree … X**

**Response:**

Thank you again! We have revised them in line to your suggestions. However, we could not get this line: “**this finding is in line with what** “ in the final manuscript that was submitted after the previous revisionPlease see the amendment in the revised manuscript with track changes.

7. **References
   Line 513, reference 6: Lapolla PMA √
   Lapolla P, M.A. X**

**Line 520, reference 8: There should be full-stop after the author’s name, that is, after WHO.The days for which the literature was cited online should be written.**
Line 569, reference 24: Insert fullstop after the name of author, ie CDC.
Line 588, reference 30: There should be up to 6 authors spelt out before stating et al.

**Response:**

Thank you again for your constructive comment. We have revised them according to your suggestions. However, Line 24 is moved to line 31 in the final manuscript that was submitted after the first revision and we have put the full stop after CDC in line 31. We have also included the days the literature was cited/accessed. In the previously revised manuscript, we had listed 6 authors before et al in all references that had more than 6 authors. Please see the amendment in the revised manuscript with track changes.

B. **Comments from Reviewer 2**
1. **Conclusion**

**Please rephrase this statement "escalate pieces of training on COVID-19 preventive measures" ('escalate' as a word in that context seems inappropriate)
Tables and Figures:**

**Response:**

Thank you again for your constructive comment. We have revised it according to your suggestions. Please see the amendment in the revised manuscript with track changes.

2. **Tables and figures**

**In the tables S3, S4 and S5. there were no percentages (in brackets) seen besides theirrespective counts/frequencies. Conventionally contingency tables should be presented first descriptively before being reported inferential or predictive. The authors should quickly rectifythis. Kindly compare them to table S6 which was better formatted/done (there will be no need for the total column in S3,S4 and S5). Figure 3 is better understood as a table.**

**Response:**

Thank you again for your constructive comment. We have revised the supplement tables and figure 3 according to your suggestions. We have included percentage column in the tables S3, S4 nd S5. We have also changed the figure-3 to a table and incorporated it into table 1. Please see the amendment in the revised manuscript with track changes.
